# Localization of PPM1H phosphatase tunes Parkinson’s disease-linked LRRK2 kinase-mediated Rab GTPase phosphorylation and ciliogenesis

**DOI:** 10.1073/pnas.2315171120

**Published:** 2023-10-27

**Authors:** Wondwossen M. Yeshaw, Ayan Adhikari, Claire Y. Chiang, Herschel S. Dhekne, Paulina S. Wawro, Suzanne R. Pfeffer

**Affiliations:** ^a^Department of Biochemistry, Stanford University School of Medicine, Stanford, CA 94305-5307; ^b^Aligning Science Across Parkinson’s Collaborative Research Network, Chevy Chase, MD 20815

**Keywords:** Parkinson’s disease, LRRK2 kinase, Rab GTPase, primary cilia, phosphatase

## Abstract

PPM1H phosphatase reverses Parkinson’s disease-associated, Leucine Rich Repeat Kinase 2-mediated Rab GTPase phosphorylation. We show here that PPM1H relies on an N-terminal amphipathic helix for Golgi localization. The amphipathic helix enables PPM1H to bind to liposomes in vitro, and small, highly curved liposomes stimulate PPM1H activity. We artificially anchored PPM1H to the Golgi, mitochondria, or mother centriole. Our data show that regulation of Rab10 GTPase phosphorylation requires PPM1H access to Rab10 at or near the mother centriole. Moreover, poor colocalization of Rab12 explains in part why it is a poor substrate for PPM1H in cells but not in vitro. These data support a model in which localization drives PPM1H substrate selection and centriolar PPM1H is critical for regulation of Rab GTPase-regulated ciliogenesis. Moreover, Golgi localized PPM1H may maintain active Rab GTPases on the Golgi to carry out their nonciliogenesis-related functions in membrane trafficking.

## Significance

Pathogenic, hyperactive LRRK2 (Leucine Rich Repeat Kinase 2) kinase is strongly linked to Parkinson’s disease and LRRK2 phosphorylates a subset of Rab GTPases that are master regulators of membrane trafficking. PPM1H phosphatase specifically dephosphorylates Rab8A and Rab10, the major LRRK2 substrates. Here, we provide unique cell biological and biochemical insight related to the localization and activation of PPM1H phosphatase. Understanding how PPM1H modulates LRRK2 activity is of fundamental interest and also important, as activators of PPM1H may eventually benefit Parkinson’s disease patients.

Hyperactive, pathogenic mutations in Leucine Rich Repeat Kinase 2 (LRRK2) represent the most common cause of inherited Parkinson's disease (1). Phosphoproteomics revealed that a subset of Rab GTPases is selected for LRRK2 action in cells (2, 3), especially Rab10 and Rab8A. Rab phosphorylation occurs within the so-called Switch 2 region that is critical for Rab effector protein recognition, guanine nucleotide exchange factor activation, and GDI-mediated recycling (4). Thus, Rab phosphorylation blocks the abilities of certain Rab GTPases to be activated and to bind to many of their cognate effector proteins (2); it also entraps them on the compartment upon which they are phosphorylated because they cannot bind their recycling chaperone, GDI (2, 5).

Despite the loss of binding capacity to many effector proteins, phosphorylated Rab proteins gain phospho-specific binding capacity to a new set of proteins including RILPL1, RILPL2 and MyoVa and LRRK2 (3, 6, 7). In particular, binding of phosphoRab10 to RILPL1 is necessary and sufficient for LRRK2 blockade of primary cilia formation (8). LRRK2-generated phosphoRab10-RILPL1 and phosphoRab10-MyoVa complexes block primary cilia formation by a mechanism upstream of recruitment of TTBK2 kinase to the mother centriole (6, 9). Pathogenic LRRK2 also enhances cilia loss by a yet-to-be-determined, Rab10 and RILPL1-independent pathway (9). Importantly, only a few percent of the total pool of Rab proteins is phosphorylated at steady state (2, 10), yet this pool is sufficient to block cilia formation in a dominant manner (3, 8).

Rab GTPase phosphorylation turns over extremely rapidly: treatment of cultured cells with LRRK2 inhibitors leads to complete dephosphorylation within just a few minutes (10). Elucidating how phosphatases control Rab phosphorylation is thus critical to our understanding of the consequences of LRRK2-mediated Rab phosphorylation. Berndsen et al. (11) reported the discovery that PPM1H is a Rab-specific phosphatase that can reverse LRRK2-mediated Rab phosphorylation. Multiple lines of evidence confirm PPM1H's role in phosphoRab biology. Loss of PPM1H phenocopies hyperactivation of LRRK2 in cell culture (11) and mouse brain (12). Moreover, after addition of LRRK2 inhibitors, cells lacking PPM1H recover Rab phosphorylation at about half the rate of control cells. Finally, unbiased mass spectrometry of proteins bound to a substrate-trapping PPM1H mutant showed strong enrichment for Rab10, Rab8A, and Rab35 (11).

We showed previously that exogenously expressed PPM1H is localized primarily to the Golgi complex, with additional localization to cytosolic and mitochondria-associated pools (11). Here, we investigate the molecular basis for PPM1H Golgi localization and probe the importance of PPM1H localization on Rab10 and Rab12 phosphorylation and function.

## Results and Discussion

**Fig. 1.**
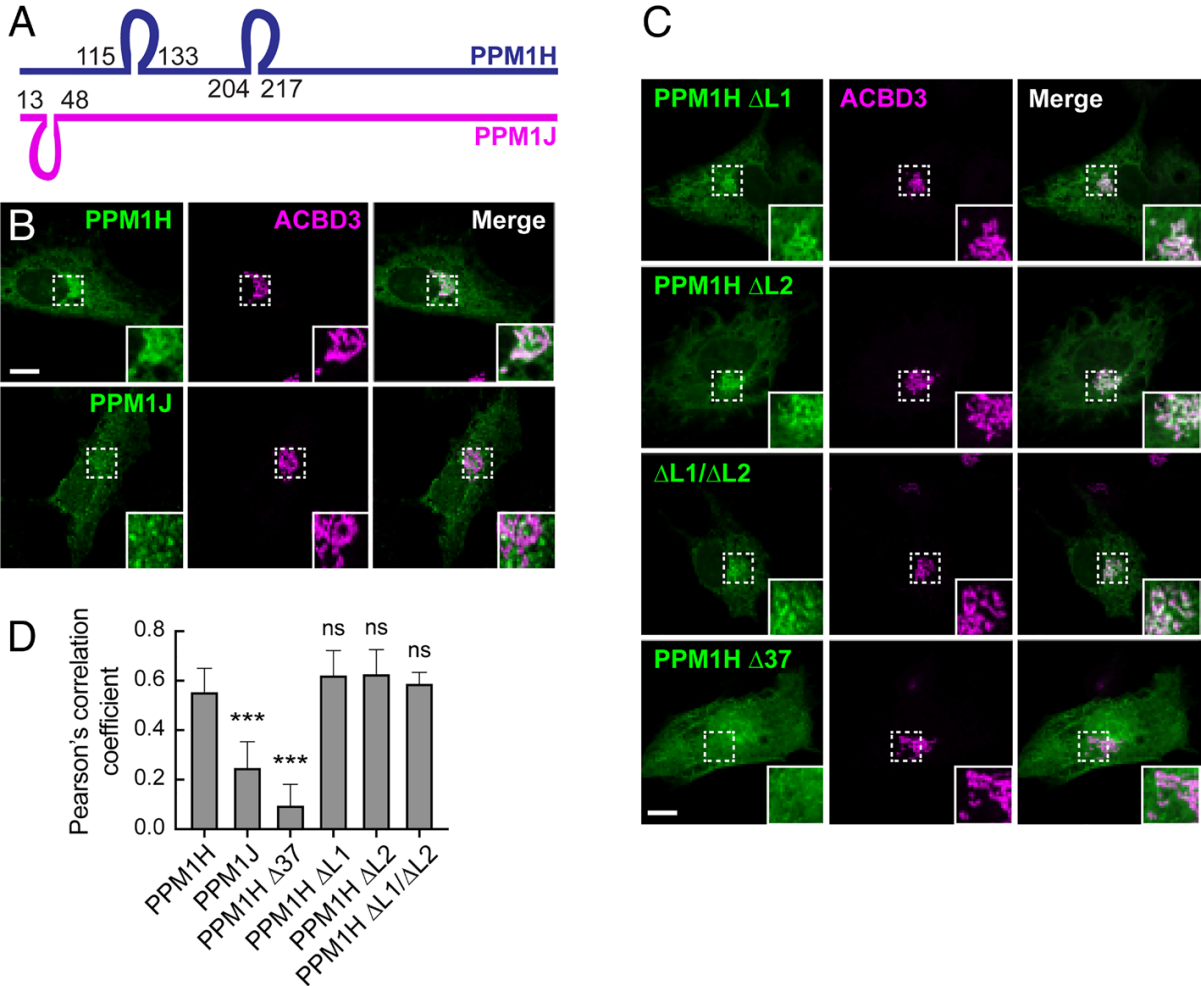
PPM1H is localized to the Golgi via its N-terminus. (*A*) Diagram of sequence alignment between PPM1H (blue) and PPM1J (magenta). The loops represent sequences that are present in one of the proteins and not related to those in the other. (*B* and *C*) Immunofluorescence microscopy of RPE cells transiently transfected with plasmids encoding either HA-PPM1H, HA-PPM1J, HA-Δ37-PPM1H, HA-ΔLoop1-PPM1H (missing residues 115 to 133), HA-ΔLoop2-PPM1H (missing residues 204 to 217), or HA-ΔLoop1/2-PPM1H (missing both loops). After 24 h, cells were fixed and stained with mouse anti-HA antibody (green) and with rabbit anti-ACBD3 to label the Golgi (magenta). (Scale bar, 10 µm.) Shown are maximum intensity projections. Areas boxed with dashed lines are enlarged at lower right. (*D*) Colocalization of PPM1H or PPM1J with ACBD3 was determined by Pearson’s coefficient. Error bars represent SEM from two independent experiments counting at least 20 cells per condition. Significance was determined relative to HA-PPM1H by one way ANOVA. In relation to HA-PPM1H, *P* values were ****P < 0.0001 for HA-PPM1J, ****P < 0.0001 for HA-Δ37 PPM1H, *P* = 0.084373 for HA-ΔLoop1-PPM1H, *P* = 0.060330 for HA-ΔLoop2-PPM1H, and *P* = 0.421158 for HA-ΔLoop1/2-PPM1H.

To identify the portion of PPM1H responsible for its Golgi localization, we aligned the PPM1H sequence and full length, predicted Alphafold structure with that of its nearest relative, PPM1J that is not Golgi localized (Fig. 1*A*, *SI Appendix*, Fig. S1, and Fig. 1 *B* and *D*). The two proteins are highly structurally homologous but contain distinct loop sequences more readily compared using a linear schematic (Fig. 1*A*). Amino acid insertions between PPM1H residues 115 to 133 and 204 to 217 distinguish the PPM1H sequence from that of PPM1J; similarly, PPM1J’s amino terminal region contains an insert between residues 13 and 48 in comparison with PPM1H (Fig. 1*A*). Analysis of mutant PPM1H proteins missing either or both of these loops revealed that both were dispensable for PPM1H colocalization with the Golgi marker, ACBD3 (Fig. 1*C*). In contrast, removal of the N-terminal 37 residues (Δ37) led to a cytosolic distribution of PPM1H protein (Fig. 1 *C* and *D*); the hazy staining PPM1J was detected in small structures in the perinuclear region that lacked the cisternal appearance of Golgi-associated ACBD3 and may represent late endosomes (Fig. 1*B*). Costaining of cells expressing PPM1H and PPM1J with the mitochondrial marker, mitofilin, confirmed that most of the proteins were not mitochondrially associated (*SI Appendix*, Fig. S2 *A* and *B*); nevertheless, a small proportion of PPM1H colocalized with mitofilin as we have reported previously (11). PPM1H has also been reported to be phosphorylated (13); nonphosphorylatable S124A and S211A PPM1H proteins were not altered in their localizations (*SI Appendix*, Fig. S3).

### Golgi Surveillance by PPM1H Phosphatase.

**Fig. 2.**
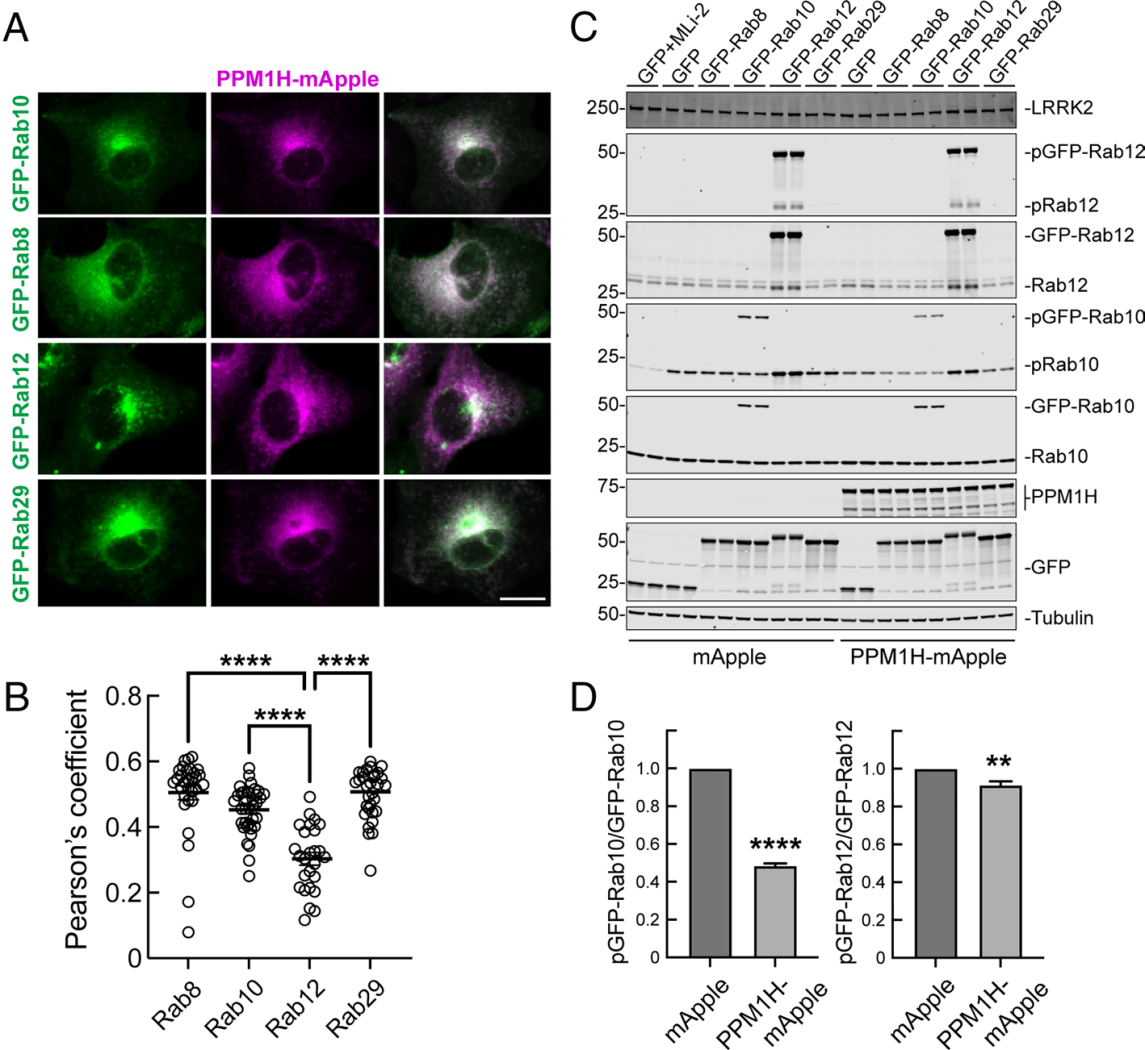
Localization of endogenous Rabs in A549 cells. (*A*) A549 cells were transduced with lentiviruses encoding PPM1H-mApple (magenta) and either GFP-Rab8A, GFP-Rab10, GFP-Rab12, or GFP-Rab29 (green) for stable overexpression. (Scale bar, 10 µm.) Representative images are shown as maximum intensity projections. (*B*) Colocalization of Rabs with PPM1H was determined by Pearson’s coefficient. Error bars represent SEM from two independent experiments with >20 cells per condition. Significance was determined relative to GFP-Rab12 by one way ANOVA. ****P < 0.0001. (*C*) A549 cells transduced with lentiviruses as in A and treated ±MLi-2 (200 nM for 2 h) were lysed and analyzed by immunoblotting. Proteins were detected using mouse anti-LRRK2, rabbit anti-pRab12, mouse anti-total Rab12, rabbit anti-pRab10, mouse anti-total Rab10, rabbit anti-PPM1H, chicken anti-GFP, and mouse anti-α-Tubulin antibodies. (*D*) Quantification of the ratio of pGFP-Rab10 to total GFP-Rab10 (*Left*) and pGFP-Rab12 to total GFP-Rab12 (*Right*). Error bars represent SEM from three independent experiments. Significance was determined relative to the mApple control by student’s *t* test. ****P < 0.0001 and **P = 0.0016.

Phosphatase localization can play an important role in regulation of phospho-substrate selectivity. Rab8A, 10, 12, 29, 35, and 43 are all LRRK2 substrates, and several of these are normally localized at or near the Golgi complex; Rab8A, Rab10, and Rab12 are the most predominant and ubiquitously expressed LRRK2 substrates (2, 3). We determined the localizations of GFP-tagged Rab8A, 10, 12, and 29 in A549 cells, in relation to PPM1H-mApple (endogenous PPM1H cannot be detected with current reagents). As shown in Fig. 2 *A* and *B*, exogenous PPM1H showed the highest level of colocalization with Rab8A and Rab29; Rab10 and especially Rab12 showed less similar distributions but displayed some overlap with PPM1H. We showed previously strong colocalization of HA-PPM1H with exogenous GFP-Rab10 in RPE cells (11); the extent of colocalization is somewhat cell type specific. Thus, PPM1H on the Golgi may protect Golgi-associated Rab proteins from LRRK2 phosphorylation and inactivation. There is sufficient overlap for exogenously expressed, membrane-associated PPM1H to access each of its substrates, although they may be dephosphorylated with different efficiencies, with Rab12 showing the lowest extent of colocalization (Fig. 2*B*).

It is important to note that A549 cells contain only 37,000 molecules of PPM1H in relation to 2.6 × 10^6^ molecules of Rab10, 960,000 Rab8A, 135,000 Rab12, and 25,000 Rab29 (https://copica.proteo.info/#/copybrowse/A549_single_shot.txt). Exogenously expressed PPM1H may show a broader distribution than the endogenous protein but does not influence Rab localization. In addition, we cannot distinguish whether the small percentage of a total Rab protein that is LRRK2 phosphorylated (10) has adequate access to endogenous PPM1H. Despite these limitations, Rab12 showed the least amount of colocalization with exogenously expressed PPM1H.

Immunoblot analysis of these cells confirmed that exogenous PPM1H was capable of dephosphorylating phosphoRab10 efficiently (Fig. 2*C*). Note that as we report elsewhere, expression of GFP-Rab12 activates LRRK2 kinase activity and phosphoRab GTPase levels (14). Nevertheless, phosphoRab12 was not efficiently dephosphorylated by PPM1H under these moderate expression conditions (Fig. 2 *C* and *D*).

### PPM1H’s Amphipathic Helix Is Needed for Golgi Localization and Best Activity.

**Fig. 3.**
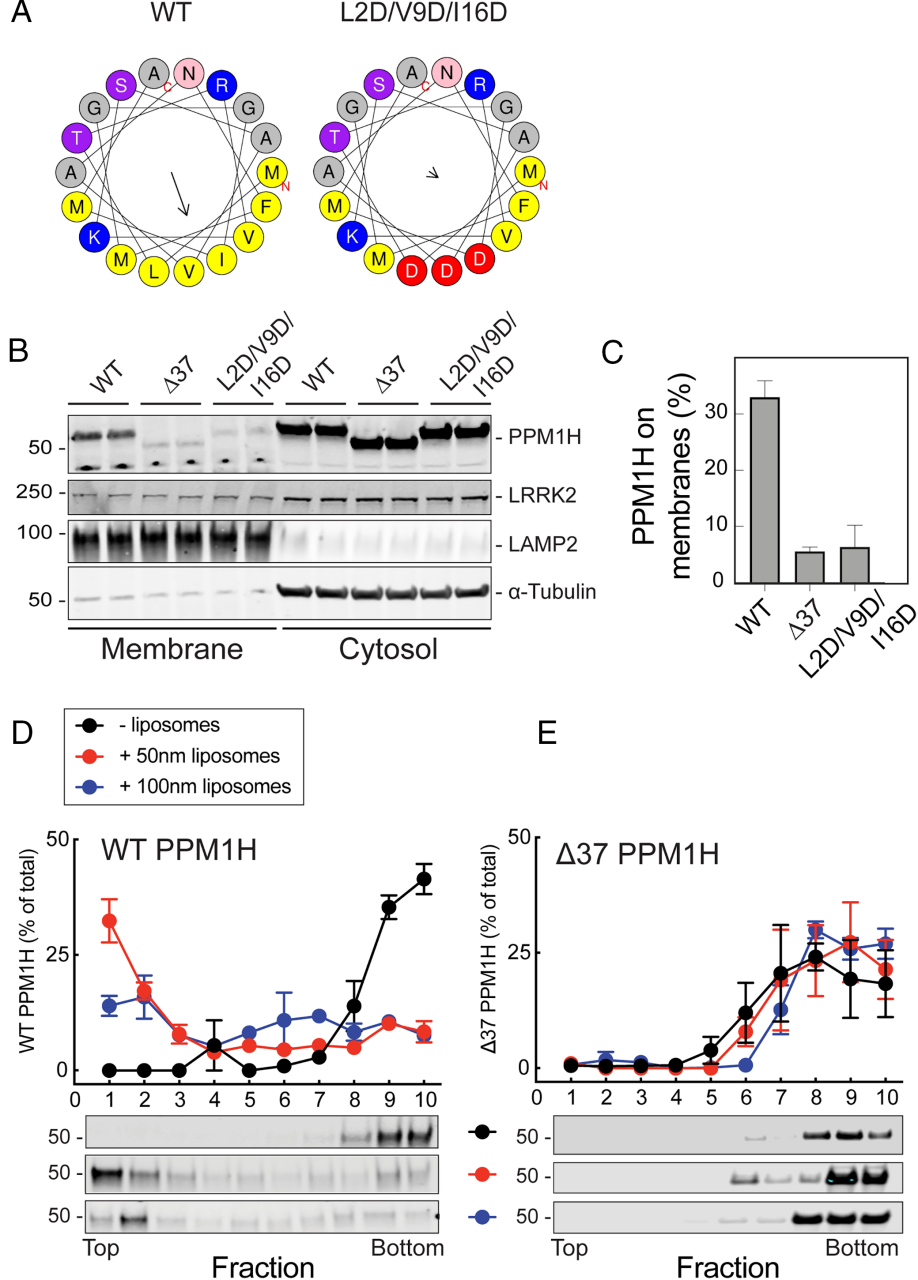
PPM1H’s N-terminal amphipathic helix is needed for membrane association. (*A*) Helical wheel projections made with Heliquest software (17) revealing the amphipathic helix at the N-terminus of PPM1H and location of L2D/V9D/I16D mutant residues introduced within the helix to block amphipathicity. The arrowheads represent the hydrophobic moment of the helix; the length of the arrow indicates the degree of hydrophobicity. (*B* and *C*) HEK293T cells were transiently transfected with plasmids encoding either HA-PPM1H, HA-Δ37-PPM1H, or HA-L2D/V9D/I16D-PPM1H. After 36 h, cells were harvested and membrane and cytosol fractions obtained. Then, 80 µg membrane protein and an equivalent volume of cytosolic fractions were processed for immunoblotting. Proteins were detected using mouse anti-LRRK2, mouse anti-LAMP2, mouse anti-HA, mouse anti alpha-tubulin antibodies. (*C*) Quantification of the fraction of HA-PPM1H on membranes. Error bars represent SD from two independent experiments. (*D* and *E*) Sucrose gradient flotation of wild type (*D*) or Δ37 PPM1H (*E*) in the presence of 50 nm or 100 nm liposomes. The distribution of PPM1H across the gradient was determined by immunoblot; fractions were collected from the top. Quantitation of three independent experiments is shown (±SEM).

Analysis of the secondary structure of the PPM1H N-terminus revealed that it comprises an amphipathic helix, with Leu2, Val9, and Ile16 lying at the center of a possible hydrophobic face (Fig. 3*A*). This was reminiscent of work from Antonny and colleagues who showed that the Golgi-associated ArfGAP1 protein relies on an amphipathic helix to associate with the Golgi; this sequence is sufficient to drive liposome association and catalytic activation of ArfGAP1 protein (15, 16).

To test the importance of the amphipathic nature of this sequence, we compared the subcellular fractionation properties of wild-type, Δ37, and L2D/V9D/I16D protein where the mutations disrupt the amphipathicity of the N-terminus (Fig. 3*A*). Upon exogenous PPM1H expression and differential centrifugation to separate cytosolic from membrane-associated proteins, ~33% of PPM1H cosedimented with membranes, compared with only ~6% of Δ37 or L2D/V9D/I16D mutant PPM1H proteins (Fig. 3 *B* and *C*). Under these conditions, ~10% of LRRK2 was membrane associated (8). These experiments demonstrate the importance of PPM1H's N-terminal 37 residues for membrane association in cells, and show that residues 2, 9, and 16 are critical for membrane association—consistent with their role in stabilizing an amphipathic helix.

Analogous to ArfGAP1, PPM1H’s N-terminus was sufficient to enable purified PPM1H to associate with liposomes as monitored by sucrose gradient flotation (Fig. 3 *D* and *E*). In these experiments, purified PPM1H or Δ37 PPM1H were incubated together with liposomes of mammalian cell Golgi lipid composition, overlaid with sucrose, and then ultracentrifuged to equilibrium. Full-length PPM1H floated to the top of the gradient in the presence of 50 nm liposomes while Δ37 PPM1H did not. Interestingly, a smaller amount of PPM1H floated to the top of gradients in the presence of less highly curved, 100 nm liposomes—in this case, a greater proportion of PPM1H was detected across the entire gradient, consistent with weaker affinity for the floating liposomes. These data confirm that the N-terminal amphipathic helix drives PPM1H membrane association in vitro, with preference for more highly curved membranes, and in cells.

### Membrane-Associated PPM1H Is Most Active.

**Fig. 4.**
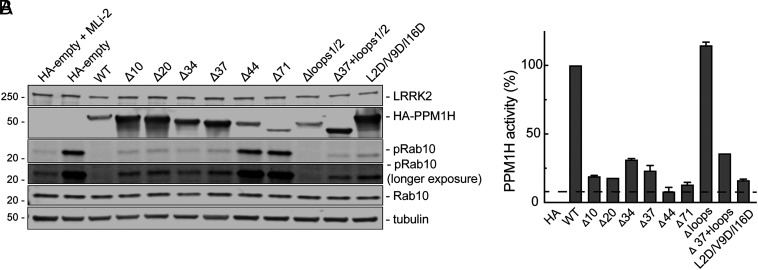
PPM1H’s amphipathic helix is needed for full activity in cells. (*A*) HEK293T cells were transiently transfected for 24 h with Flag-LRRK2 R1441C together with either HA-empty, HA-PPM1H, HA-Δ10-PPM1H, HA-Δ20-PPM1H, HA-Δ34-PPM1H, HA-Δ37-PPM1H, HA-Δ44-PPM1H, HA-Δ71-PPM1H, HA-ΔLoop1/2-PPM1H, HA-Δ37/ΔLoop1/2-PPM1H, or HA-L2D/V9D/I16D-PPM1H. After 24 h, cells were lysed and 30 µg protein analyzed by immunoblotting. Proteins were detected using mouse anti-LRRK2, rabbit anti-HA, rabbit anti-pRab10, mouse anti-total Rab10 and mouse anti alpha-tubulin antibodies. (*B*) Activity of PPM1H was quantified by normalizing pRab10/total Rab10 levels relative to PPM1H expression, with WT PPM1H set at 100% activity. Error bars represent SD from two independent experiments.

As might be expected for an enzyme that acts on membrane-associated phosphoRab GTPases, full-length PPM1H enzyme showed the greatest activity in cells compared with truncated forms of the enzyme that lack 10, 20, 34, or 37 N-terminal residues of the amphipathic helix (Fig. 4). The activity of various PPM1H protein constructs in cells was assessed by coexpression with hyperactive, Flag-tagged LRRK2 R1441C followed by immunoblotting to monitor decreases in phosphoRab10 levels (Fig. 4).

As expected, wild-type PPM1H expression abolished detectable phosphoRab10 (Fig. 4*A*, lane 3). When normalized to the expression level of each PPM1H construct, truncations of 10, 20, 34, or 37 residues greatly decreased PPM1H activity, at least upon overexpression in HEK 293T cells (Fig. 4*B*). Longer N-terminal truncations of 44 or 71 residues completely blocked PPM1H activity, confirming prior work from Waschbüsch et al. (18). Loss of the amphipathic helix by insertion of charged residues (L2D/V9D/I16D) into the predicted face of the helix also eliminated most PPM1H activity in this overexpression paradigm (Fig. 4 *A* and *B*). No activity decrease was seen for PPM1H in which the loops at positions 115 to 133 and 204 to 217 (loops 1/2) were deleted (Fig. 4). Mutation of the potential S124 and S211 phosphorylation sites was also without consequence (*SI Appendix*, Fig. S4).

**Fig. 5.**
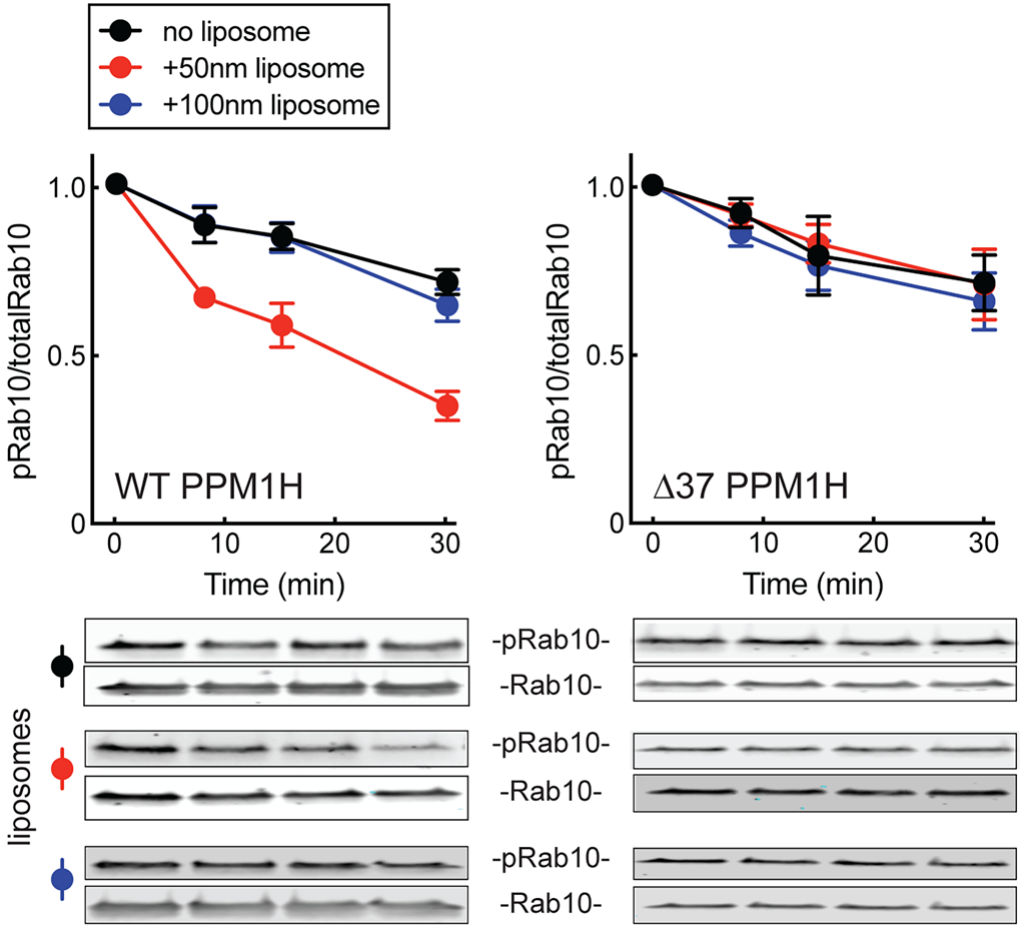
Activation of PPM1H by highly curved membranes requires its amphipathic helix. Purified PPM1H (*Left*) or Δ37 PPM1H (*Right*) was assayed for phosphatase activity in the presence of 50 nm or 100 nm liposomes for the indicated times. The respective decreases in phosphoRab10 reflect phosphatase activity. Error bars represent SEM from three independent experiments; representative gels are shown. Black circles, no liposomes; red circles, 50 nm liposomes; blue circles, 100 nm liposomes.

These experiments suggest that membrane association increases PPM1H’s activity in cells. Consistent with this possibility, highly curved 50 nm liposomes directly activated purified PPM1H enzyme in vitro. Shown in Fig. 5 is the loss of phosphoRab10 protein as a function of time in the presence of purified, full-length PPM1H protein. Here, we employed bacterially expressed and purified, soluble, PPM1H and nonprenylated Rab10 that was prephosphorylated using purified MST kinase (https://doi.org/10.17504/protocols.io.bvjxn4pn).

Inclusion of highly curved, 50 nm liposomes, but not larger, 100 nm liposomes, activated PPM1H activity at least two-fold in these in vitro reactions (Fig. 5, *Left*). These experiments were carried out with extremely limiting levels of PPM1H protein (15 ng) and Δ37 PPM1H (10 ng) in reactions containing much larger amounts of in vitro phosphorylated Rab10 protein (1.5 µg).

Importantly, the activity of Δ37 PPM1H was completely unaffected by the presence of small or large liposomes (Fig. 5, *Right*). This suggests that the interaction of PPM1H's N-terminus with a highly curved membrane in some way alters its structure to activate its catalytic activity.

The crystal structure of PPM1H is missing residues 1 to 32 (18). However, the Alphafold model of full-length PPM1H shows that the N-terminal extension (*SI Appendix*, Fig. S1, red portion) has the capacity to reach directly in front of the active site (asterisk), possibly interacting with the so-called FLAP domain that is critical for substrate recognition (18; *SI Appendix*, Fig. S1). Our current working model is that the N-terminus occludes substrate binding to the active site; binding of the amphipathic helix to a highly curved membrane may release the N-terminus from the FLAP domain, permitting substrate access. Consistent with this hypothesis is the fact that purified Δ37 PPM1H is consistently about twofold more active than wild-type PPM1H in vitro. Altogether, these data support the conclusion that membrane-associated PPM1H is more active than cytosolic PPM1H protein.

### Relocalization of PPM1H Reveals Its Potency at the Mother Centriole.

A phosphatase that is partly cytosolic should theoretically have access to Rab GTPases wherever they are located in cells. However, if membrane localization is truly important for substrate access or activation, artificial anchoring of PPM1H in different locations should provide important clues to the significance of its endogenous localization. Thus, we relocalized PPM1H to distinct cellular compartments and monitored the consequences for phosphoRab10 levels and for primary cilia formation that is a highly sensitive measure of PPM1H activity (11).

**Fig. 6.**
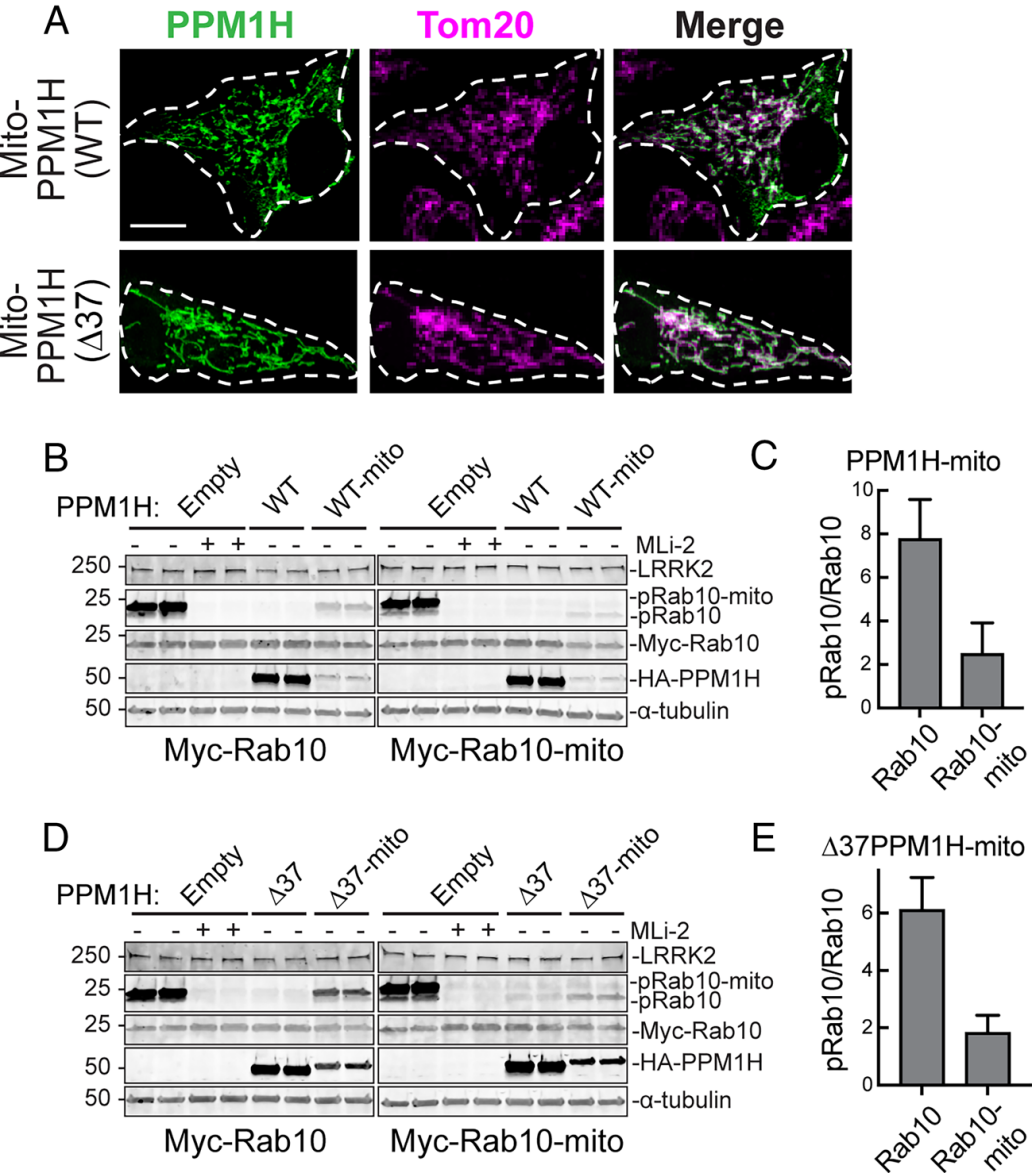
Mitochondrially localized PPM1H is catalytically active. (*A*) Representative localizations of PPM1H-mito and Δ37-PPM1H-mito proteins in RPE cells. Magnification bar, 10 µm. (*B* and *C*) Dephosphorylation of wild-type or mitochondrially targeted Myc-Rab10 by wild-type or mitochondrially targeted PPM1H in HEK293T cells as indicated. Quantitation shown in *C* and *E* represent the mean of two independent experiments. Error bars represent SD. (*D* and *E*) Same as in *B* and *C* using Δ37 PPM1H or Δ37 PPM1H-mito tag. Quantitation of changes in mito-pRab10 was carried out by analyzing only the upper mito-pRab10 band.

PPM1H was first relocalized to mitochondria by attaching an amphipathic helix derived from monoamine oxidase (19) to the PPM1H C-terminus; this hybrid protein will be referred to as mito-PPM1H. Note that the mitochondrial targeting signal dominated the targeting process when added to either full-length PPM1H protein or Δ37 PPM1H (Fig. 6*A*).

We next explored the ability of wild-type or mitochondrially anchored PPM1H to dephosphorylate Rab10 or mitochondrially anchored Rab10. In these experiments, the PPM1H was much more highly expressed than the mitochondrially anchored protein (Fig. 6 *B* and *D*) but comparisons could nevertheless be made between those amounts of mito-PPM1H acting on either wild-type or mitochondrially anchored Rab10 proteins. Not surprisingly, mitochondrially anchored PPM1H proteins (mito-PPM1H or Δ37 mito-PPM1H) were better able to dephosphorylate mitochondrially anchored Rab10 protein than wild-type Rab10 (Fig. 6 *C* and *E*). Wild-type (Fig. 6*B*) and Δ37 PPM1H (Fig. 6*D*) efficiently dephosphorylated wild-type and mitochondrially anchored Rab10 at these expression levels, presumably due to diffusion. These experiments confirm catalytic activity of mitochondrially localized PPM1H forms.

**Fig. 7.**
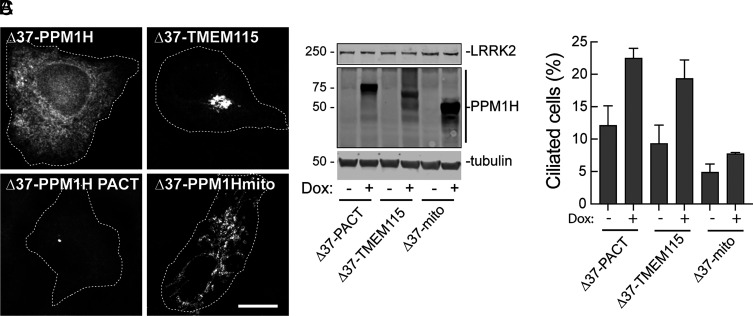
PPM1H influences ciliogenesis from the mother centriole or Golgi but not mitochondria. (*A*) PPM1H KO A549 cells were transduced with lentiviruses encoding HA-tagged Dox-inducible Δ37-PPM1H, Δ37-PPM1H-PACT, Δ37-PPM1H-TMEM115, and Δ37-PPM1H-mito. HA-PPM1H signal was detected by using mouse anti-HA antibody. Shown are the localizations of indicated constructs. Magnification bar, 10 µm. (*B*) Immunoblot of construct expression for the experiments presented in *A*. Proteins were detected using mouse anti-LRRK2, mouse anti-HA, and mouse anti alpha-tubulin antibodies. (*C*) Percent ciliated cells with or without addition of 1 µg/mL DOX for 24 h to induce expression of the indicated constructs. Shown is the mean of two independent experiments. For the PACT construct, only cells displaying uniquely centrosomal PPM1H localization were scored. Error bars represent SD from two independent experiments with >300 cells per condition.

We also anchored PPM1H stably on the Golgi complex and at the mother centriole by appending sequences from TMEM115 (Golgi) or the PACT domain (centriole). As shown in Fig. 7*A*, Δ37 PPM1H represented the cytosolic form; Δ37 TMEM115 PPM1H was exclusively Golgi localized, Δ37 PPM1H PACT was mostly centriolar but some cells also showed a broader cytoplasmic distribution; Δ37 PPM1H-mito was constrained to mitochondria. The expression of each of these constructs was under Doxycycline (DOX) control, and addition of DOX turned on the expression of proteins of the correct molecular mass (Fig. 7*B*).

Using this sensitive and controlled expression system, Golgi and centriole-anchored PPM1H were both able to greatly stimulate the percentage of A549 cells that were ciliated, presumably due to decreased phosphoRab10 levels. This was in contrast with cells expressing much higher levels of mito-PPM1H. This experiment shows that PPM1H localized to the centriole or Golgi region can access and act upon phosphoRabs to permit primary cilia formation.

### PPM1H Acts on phosphoRab12 In Vitro but Not In Vivo.

As mentioned above, previous work showed that phosphoRab8A and phosphoRab10 are much better PPM1H substrates than phosphoRab12 in cells [Berndsen et al. (11); see also Fig. 2]. Moreover, a PPM1H substrate-trapping mutant failed to trap Rab12 in cells as determined by mass spectrometry. Thus, in vitro dephosphorylation experiments have focused on phosphoRab8A and phosphoRab10 (11, 18). Given the proportionally lower extent of colocalization of Rab12 with PPM1H in relation to Rab8A and Rab10, it was possible that phosphoRab12 is a much poorer substrate for PPM1H in cells simply because the two proteins colocalize less well (Fig. 2). Alternatively, PPM1H enzyme may discriminate between Rab substrates and simply prefer Rab8A and Rab10.

**Fig. 8.**
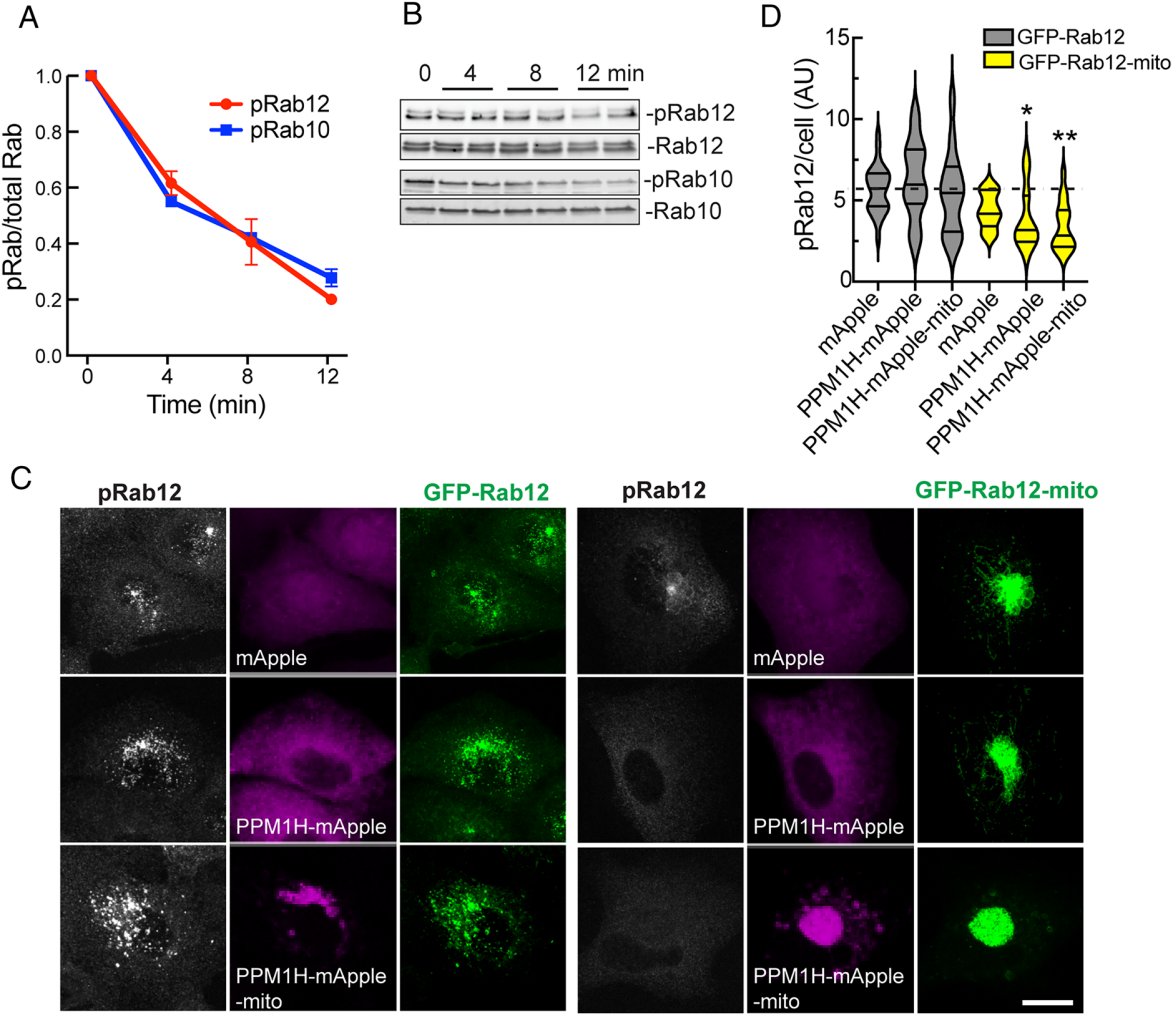
Localization is an important determinant of pRab12 dephosphorylation by PPM1H. (*A*) Purified PPM1H was assayed for phosphatase activity with purified pRab12 or pRab10 in the presence of 50 nm liposomes for the times indicated. Phosphatase activity was monitored by immunoblot (as in *B*) to detect loss of pRab12 or pRab10 as a function of time. Shown are the means of two independent experiments carried out in duplicate, error bars represent SD. (*C*) A549 cells were transduced with lentiviruses encoding mApple (magenta), PPM1H-mApple, or PPM1H-mApple-mito and either GFP-Rab12 (green) or GFP-Rab12-mito to create stably expressing cell lines. Cells were subsequently stained with anti-pRab12 antibody (white). (Scale bar, 10 µm.) Representative images are shown as maximum intensity projections. (*D*) Shown is the amount of pRab12 per cell quantified as a mean integrated density. Error bars represent SEM from three independent experiments with at least 30 cells per condition. Significance was determined relative to values in GFP-Rab12-mito cells expressing mApple by *t* test; *P = 0.0424 for PPM1H-mApple, *P = 0.0369 for PPM1H-mApple-mito.

We tested the ability of purified PPM1H to dephosphorylate phosphoRab10 and phosphoRab12 to distinguish between these possibilities. Fig. 8 shows the kinetics of PPM1H-mediated Rab10 and Rab12 dephosphorylation: both Rabs were dephosphorylated at the same rate in vitro when assayed in parallel with limiting amounts (25 ng) of purified enzyme. Thus, substrate selectivity cannot explain the inefficient dephosphorylation of phosphoRab12 in cells.

To further evaluate the importance of localization on PPM1H substrate selectivity, we relocalized both Rab12 and PPM1H to mitochondria and monitored the ability of PPM1H to dephosphorylate Rab12. Fig. 8 *C* (*Left*) shows the localizations of GFP-Rab12, phosphoRab12, and PPM1H in cells expressing mApple, PPM1H mApple, or mitochondrially anchored PPM1H mApple. In all cases, phosphoRab12 levels were unchanged (Fig. 8 *C* and *D*). In contrast, when Rab12 was localized to mitochondria, it was LRRK2 phosphorylated to a small extent, and was a much better substrate for mitochondrially anchored PPM1H enzyme (Fig. 8 *C* and *D*). Wild-type PPM1H also acted a little bit on mitochondrially anchored Rab12 protein; we believe that this is most likely due to the small amount of mitochondrially localized wild-type PPM1H protein (*SI Appendix*, Fig. S2). These experiments strongly suggest that Rab12 localization explains why it is a poor PPM1H substrate relative to Rab8A and Rab10 proteins in cells; if it was somehow protected from PPM1H action due to effector binding, that effector should have been able to access phosphoRab12 on mitochondrial surfaces. Indeed, mitochondrially targeted Rab12 causes collapse of the mitochondria into a bundle; this is likely due to an effector interaction but it does not impede PPM1H action.

## Summary

We have shown here that PPM1H is a predominantly Golgi-localized enzyme with high colocalization with Rab8A, Rab10, and Rab29 in A549 cells and with GFP-Rab10 in RPE cells. This localization is consistent with PPM1H’s role as a phosphoRab GTPase-specific phosphatase (11). Membrane association of PPM1H in cells and in vitro is driven by the protein’s N-terminal 37 residues that are predicted to form an amphipathic helix. Indeed, these residues drive liposome association with preference for small liposomes that have a high degree of membrane curvature. This is reminiscent of ArfGAP1, a Golgi associated enzyme that is important for COP-I vesicle formation and is activated on highly curved membranes via an internal amphipathic helix (15, 16).

High membrane curvature occurs at the rims of the Golgi complex and proteins containing amphipathic helices may accumulate in that subdomain. Importantly, the ciliary pocket at the base of the primary cilium is also highly curved and an intriguing possibility is that endogenous PPM1H becomes concentrated there. Unfortunately, it is impossible to determine the localization of endogenous PPM1H protein as it occurs in cells at ~37,000 copies in relation to millions of Rab8A and Rab10 proteins and could not be reliably detected by a recently available commercial antibody (AbCam EPR26028-53) when knockout (KO) cells were compared. Nevertheless, siRNA depletion of the few copies of PPM1H in wild-type mouse embryonic fibroblasts is sufficient to block ciliogenesis, demonstrating a normal role of LRRK2-mediated Rab phosphorylation in regulating ciliogenesis (11).

PPM1H is likely to work in proximity with LRRK2 on membrane surfaces, as Rab phosphorylation shows rapid turnover and is rapidly reversed upon addition of kinase inhibitors (10, 11). In this regard, it is interesting to consider the possibility that lysosome-associated LRRK2 hyperactivation upon lysosomal membrane damage (20, 21) is exacerbated by the possible absence of PPM1H at that location. In addition, PPM1H alone cannot explain the reversal of Rab GTPase phosphorylation: PhosphoRab12, for example, is a poor PPM1H substrate and is likely a substrate for another phosphatase. Future work will elaborate the full panel of phosphatases that balance the action of LRRK2 kinase on Rab GTPase phosphorylation.

## Materials and Methods

### Cloning and Plasmids.

DNA constructs were amplified in *Escherichia coli* DH5α or STBL3 and purified using mini prep columns (Econospin). DNA sequence verification was performed by Sequetech (http://www.sequetech.com). pET15b Rab10 Q68L 1-181 and pET15b His-Mst3 were kind gifts of Amir Khan (Harvard University). His-SUMO PPM1H full length, 38 to 514 were all cloned from pCMV5D HA-PPM1H into pET15b backbone. His SUMO Rab10 (1 to 181) Q68L and His SUMO Rab12 (Q101L) were cloned from pET15b Rab10 (1 to 181) Q68L and pQE 80L Rab12 (Q101L) respectively into pET15b backbone. pCMV5D HA-PPM1J (DU68077) and pCMV5D HA-PPM1H (DU62789) were obtained from the MRC-PPU Dundee. HA-PPM1H Δ10, Δ20, Δ34, Δ37, Δ44, Δ71 were all cloned from pCMV5D HA-PPM1H by PCR. PPM1H phosphorylation site mutants (S124A, S211A, S124A/S211A) were cloned from pCMV5D HA-PPM1H by site-directed mutagenesis using Q5 polymerase (New England Biolabs). To generate mitochondrially targeted PPM1H, pCMV5D HA-PPM1H was first PCR amplified to contain an EcoRI site using 5′-GGGTAAGCGGCCGCTGATGACAGCTTGTTTCC-3′ and 5′-ACGCCTAAGAATTCGTCGAGTCTAGAGGGCCCGTT-3′ primers. A mitochondrial targeting sequence was PCR amplified from mito-Rab29 (5) using 5′-GGTGCGGCCGCCTTCTGGGAAAGGA-3′ and 5′-GAATAGGGGAATTCCCCTCAAGACCGTGGCAGGAG-3′ primers and added to the C-termini of HA-PPM1H or Δ37 HA-PPM1H between the NotI and EcoRI sites. To generate PPM1H-mApple-mito and GFP-Rab12-mito we used Gibson assembly in the CSII-PPM1H-mApple and pMCB306-GFP-Rab12 backbone (14). A detailed protocol can be found here https://doi.org/10.17504/protocols.io.eq2lyjwyqlx9/v1.

### Cell Culture and Lysis.

A549, RPE, and HEK293T cells were purchased from ATCC. PPM1H KO A549 cells were obtained from MRC-PPU. Cells were cultured in DMEM containing 10% (v/v) fetal calf serum, 2 mM L-glutamine, 100 U/mL penicillin, and 100 µg/mL streptomycin. All cells were grown at 37 °C, 5% CO_2_ in a humidified atmosphere and regularly tested for *Mycoplasma* contamination. Unless otherwise indicated, cells were lysed in an ice-cold lysis buffer containing 50 mM Tris–HCl, pH 7.4, 1% (v/v) Triton X-100, 10% (v/v) glycerol, 0.15 M NaCl, 1 mM sodium orthovanadate, 50 mM NaF, 10 mM 2-glycerophosphate, 5 mM sodium pyrophosphate, 1 µg/mL microcystin-LR, and complete EDTA-free protease inhibitor cocktail (Roche). Lysates were clarified by centrifugation at 10,000g at 4 °C for 10 min and supernatant protein was quantified by Bradford assay. Detailed methods for cell transfection and cell lysis can be found in https://doi.org/10.17504/protocols.io.bw4bpgsn.

### Protein Purification.

His-SUMO-PPM1H and His-SUMO Δ37 PPM1H were purified according to this protocol https://doi.org/10.17504/protocols.io.bvjxn4pn. The purified proteins were dialyzed overnight with Ulp1 SUMO protease (100 ng/1 mg of protein) to remove the His-SUMO tag from PPM1H. The dialyzed proteins were collected the next day and concentrated before application onto an Superdex™ 75 Increase 10/300 GL (Cytiva #29148721) column fitted with 1 mL HiTrap TALON crude column (Cytiva #28953766) to remove the uncleaved His SUMO protein and the His-tagged Ulp1 SUMO protease.

### 293T Overexpression Assays of PPM1H Mutants.

HEK293T cells were seeded into six-well plates and transiently transfected at 60 to 70% confluency using polyethylenimine (PEI) transfection reagent. Then, 1 µg Flag-LRRK2 R1441C, 0.25 µg HA-PPM1H constructs, and 6.25 µg PEI were diluted in 200 µL Opti-MEM™ Reduced serum medium (Gibco™) per well. Twenty-four hours after transfection, cells were treated with 200 nM MLi-2 for 2 h as indicated and lysed in an ice-cold lysis buffer. Samples were prepared for immunoblotting analysis according to the protocol.io https://doi.org/10.17504/protocols.io.bsgrnbv6.

### Immunofluorescence Microscopy.

A549 or RPE cells were transiently transfected with HA-PPM1H plasmids. After 24 h, cells were fixed with 4% (v/v) paraformaldehyde for 10 min, permeabilized with 0.1% Triton X-100 for 5 min, and blocked with 1% BSA for 1 h (https://doi.org/10.17504/protocols.io.ewov1nmzkgr2/v1). Cells were subsequently stained with mouse or rabbit anti-HA antibody (Sigma-Aldrich H3663 or H6908, 1:1,000) and the following markers: rabbit anti-ACBD3 1:1,000 (Sigma-Aldrich HPA015594) or Rabbit anti-mitofilin 1:250 (Novus). Highly cross adsorbed H+L secondary antibodies (Life Technologies) conjugated to Alexa 488 or Alexa 568 were used at 1:5,000. Primary and secondary antibody incubations were for 1 h at room temperature (RT). Nuclei were stained with 0.1 µg/mL DAPI (Sigma). Images were obtained using a spinning disk confocal microscope (Yokogawa) with an electron multiplying charge-coupled device (EMCCD) camera (Andor, UK) and a 100x1.4NA oil immersion and a 63X glycerol immersion objectives or using a Zeiss LSM 900 microscope acquired using Zen 3.4 and a 63× objective. Images were converted to maximum intensity projections using Fiji (https://fiji.sc/) and analyzed using CellProfiler software (22, 23). Pearson’s correlation coefficients were obtained as described in https://doi.org/10.17504/protocols.io.rm7vzbqp5vx1/v1.

### Liposome Preparation.

Liposomes were generated with a bona fide mammalian cell Golgi composition of (18:0 to 20:4)PC:(18:0 to 20:4)PI:(18:0 to 18:2)PS:(18:1) plus PI(4)P:cholesterol (78:7:5:1:9) (Avanti Polar Lipids; ref. 24) for all liposome experiments except Fig. 8 *A* and *B*. In those experiments, the liposome composition was DOPC:DOPS:PI(4)P (69:30:1) but similar results were obtained with either lipid composition. A dried film of the indicated lipid mixture dissolved in chloroform was obtained by evaporation under a stream of nitrogen. The film was then resuspended in 50 mM HEPES pH 7.5,120 mM KCl. After two brief sonication cycles (5 s each) using a bath sonicator, the liposome suspension was extruded 21 times sequentially through 0.4, 0.1, and 0.05 µm pore size polycarbonate filters using a hand extruder (Avanti). The final lipid concentration in the liposome suspension was 15 mM. (A detailed protocol can be found here https://doi.org/10.17504/protocols.io.5qpvo3ke7v4o/v1).

### Sucrose Density Gradient Flotation.

Liposome flotation assays were performed following the method described by Bigay et al. (15) with slight modifications. Briefly, 1.5 mM 50 nm and 100 nm liposomes [PC:PI:PS:PI(4)P:cholesterol] were incubated with 1mM PPM1H or Δ37-PPM1H for 30 min at 30 °C in a total volume of 33 µL in HKM buffer (20 mM HEPES pH-7.5,150 mM potassium acetate, 1 mM magnesium chloride). Then, 167 μL of 60% w/v sucrose was then added and mixed to adjust the mixture to 50% sucrose. The high sucrose mixture was overlaid with 200 μL, 25% w/v sucrose and 100 μL of HKM buffer. The sample was centrifuged at 75,000 × g for 2.0 h in a Ti55 swinging bucket rotor. Ten 50 μL fractions were manually collected using a pipetman by aspiration from the top of each tube. The fractions were then analyzed by immunoblotting to detect PPM1H or Δ37 PPM1H using rabbit anti-PPM1H antibody.

### Immunoblot Determination of Membrane-Associated PPM1H.

Crude membrane fractionation was isolated according to (https://doi.org/10.17504/protocols.io.yxmvmnb99g3p/v1). Briefly, HEK293T cells were transfected with either HA-PPM1H, HA-Δ37PPM1H or HA- L2D/V9D/I16DPPM1H constructs. Thirty-six hours after transfection, cells were washed 2x with ice-cold PBS and swelled in 800 µL of hypotonic buffer (10 mM HEPES, PH = 7.4). After 20 min, 200 µL of buffer (5x) was added to achieve a final concentration of 1x resuspension buffer (50 mM HEPES pH 7.4, 150 mM NaCl, 5 mM MgCl2, 0.5 mM DTT, 100 nM GDP, 1× protease inhibitor cocktail (Sigma) and 5 mM sodium fluoride, 2 mM sodium orthovanadate, 10 mM beta-glycerophosphate, 5 mM sodium pyrophosphate, 0.1 μg/mL Microcystin-LR). The suspension was passed 20 times through a 27G needle. Lysate was spun at 1,000 g for 5 min to pellet nuclei. The postnuclear supernatant was spun at 55,000 RPM for 20 min in a tabletop ultracentrifuge in TLA100.2 rotor; the resulting supernatant was collected as cytosolic fraction. The membrane pellet was solubilized in a resuspension buffer containing 1% Triton X-100. Protein concentrations were estimated by Bradford assay (Bio-Rad, Richmond, CA). Samples containing 80 μg of membrane protein or the equivalent volume of cytosolic protein were processed for immunoblotting. All centrifugation steps were done at 4 °C. A more detailed protocol for immunoblot analysis can be found at https://doi.org/10.17504/protocols.io.bsgrnbv6.

### PPM1H Phosphatase Assay In Vitro.

Untagged Rab10 (1 to 181) Q68L or untagged Rab12 (full length) Q101L was incubated with His-MST3 kinase in a reaction buffer (50 mM HEPES pH 8,100 mM NaCl, 5 mM MgCl_2_,100 μM GTP, 0.5 mM TCEP, 10% glycerol, 5 μM BSA) at 4 °C overnight to phosphorylate Rab10/Rab12 (For a detailed protocol see https://doi.org/10.17504/protocols.io.bvjxn4pn). The next day, His-MST3 kinase was removed by passing the sample through a 1-mL syringe column containing 100 μL (50%) Ni-NTA slurry; the flow through containing phosphorylated Rab10/Rab12 was collected. In addition, 1 µg phosphorylated Rab10/Rab12 was then incubated with 15 ng PPM1H (For a detailed protocol see https://doi.org/10.17504/protocols.io.bu7wnzpe) or 10 ng Δ37 PPM1H in the presence or absence of liposomes at 30 °C. Reactions were stopped by the addition of 5X SDS-PAGE sample buffer. Samples were then analyzed by immunoblotting to detect for dephosphorylation of Rab10/Rab12 in the presence or absence of liposomes using anti-pRab10 (1:1,000) antibody or anti-pRab12 (1:1,000) antibody.

A detailed protocol for immunoblotting (25) is at https://doi.org/10.17504/protocols.io.bsgrnbv6. Then, 20 µg protein was resolved by SDS PAGE and transferred onto nitrocellulose membranes using a Bio-Rad Trans-turbo blot system. Membranes were blocked with 2% BSA in Tris-buffered saline with Tween-20 for 30 min at RT. Primary antibodies were diluted in blocking buffer as follows: mouse anti-LRRK2 N241A/34 (1:1,000, Neuromab); mouse anti-Rab10 (1:1,000, Nanotools); and rabbit anti-phospho Rab10 (1:1,000, Abcam). Primary antibody incubations were done overnight at 4 °C. LI-COR Biosciences secondary antibodies diluted in the blocking buffer were 680-nm donkey anti-rabbit (1:5,000) and 800-nm donkey anti-mouse (1:5,000). Secondary antibody incubations were for 1 h at RT. Blots were imaged using an Odyssey Infrared scanner (LI-COR) and quantified using ImageJ software (26).

### Ciliation.

PPM1H KO A549 cells were infected with lentiviruses encoding indicated constructs and on day 3, infected cells were selected using 10 µg/mL Blasticidin for 72 h as described (https://doi.org/10.17504/protocols.io.eq2ly7wpmlx9/v1); pools of stably infected cells were then assayed for cilia formation. Expression was induced using 1 µg/mL DOX for 24 h. Ciliation was monitored after 48 h serum starvation using anti-Arl13B antibody (NeuroMab, Davis, California) to stain cilia for immunofluorescence microscopy (8). Z-stack images were converted to maximum intensity projections using Fiji (https://fiji.sc/), and cilia were counted using an automated pipeline as described in detail here: https://doi.org/10.17504/protocols.io.bp2l6x2j5lqe/v1.

### Data Analysis.

Data analysis was carried out using GraphPad Prism version 9 for Mac, GraphPad Software, Boston, Massachusetts USA, www.graphpad.com. Structure models in the *SI Appendix* were generated using ChimeraX software (27).

## Supplementary Material

Appendix 01 (PDF)

## Data Availability

All primary data have been deposited in Zenodo (28).
