# Supplementary material for: Localization of PPM1H phosphatase tunes Parkinson’s disease-linked LRRK2 kinase-mediated Rab GTPase phosphorylation and ciliogenesis
Source: Proc Natl Acad Sci U S A. 2023 Oct 27;120(44):e2315171120. doi: 10.1073/pnas.2315171120 (PMC10622911; doi:10.1073/pnas.2315171120)
Supplement: Supplementary file 1 — Appendix 01 (PDF) [file pnas.2315171120.sapp.pdf]

PNAS

Supporting Information for

Localization of PPM1H phosphatase tunes Parkinson's disease-linked LRRK2 kinase-mediated Rab GTPase phosphorylation and ciliogenesis

Wondwossen M. Yeshaw, Ayan Adhikari, Claire Y. Chiang, Herschel S. Dhekne, Paulina S. Wawro, and Suzanne R. Pfeffer

Suzanne Pfeffer, Corresponding Author
Email: pfeffer@stanford.edu

This PDF file includes:

Figures S1 to S4 with legends
Key Resources Table

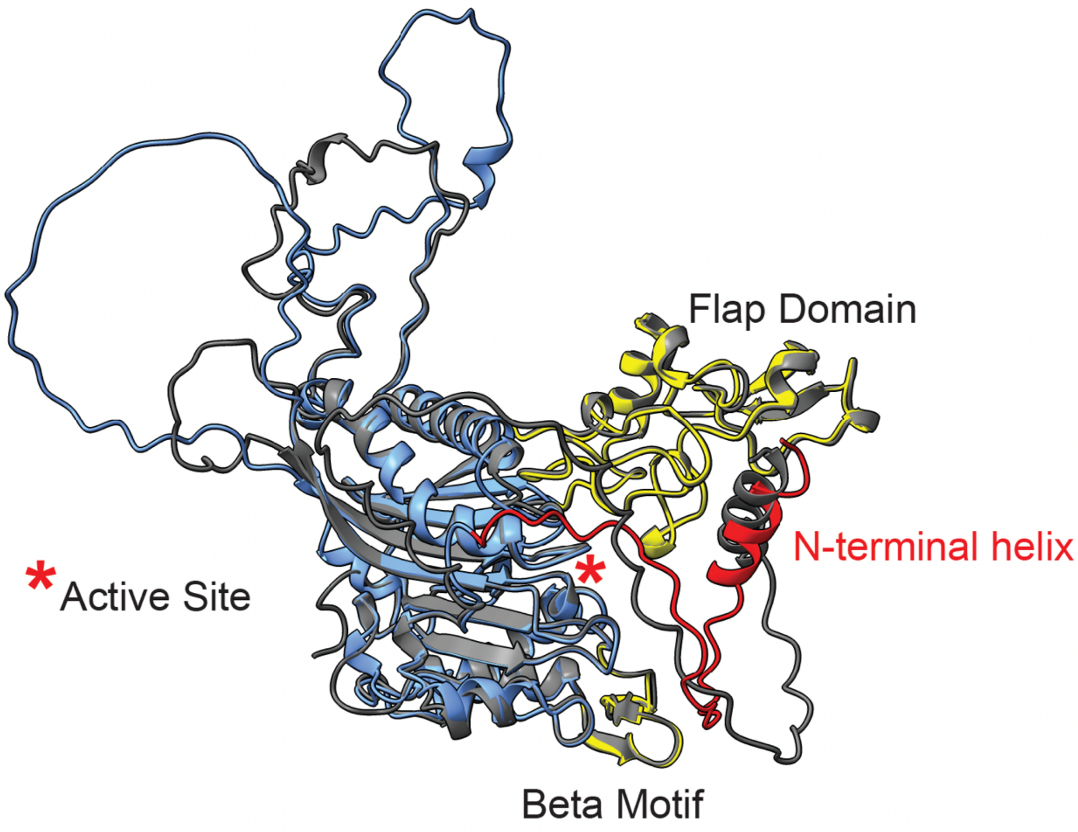

Fig. S1. Overlay of PPM1H (blue) and PPM1J (gray) Alphafold structures drawn using Chimera X software (27). The PPM1H FLAP domain and Beta motif (16) are highlighted in yellow; the PPM1H N-terminal 37 residues are shown in red. Note the perfect overlap of the core structures with loop differences, and active site occlusion by the N-termini of PPM1H and PPM1J. The location of the active site is shown as an asterisk.

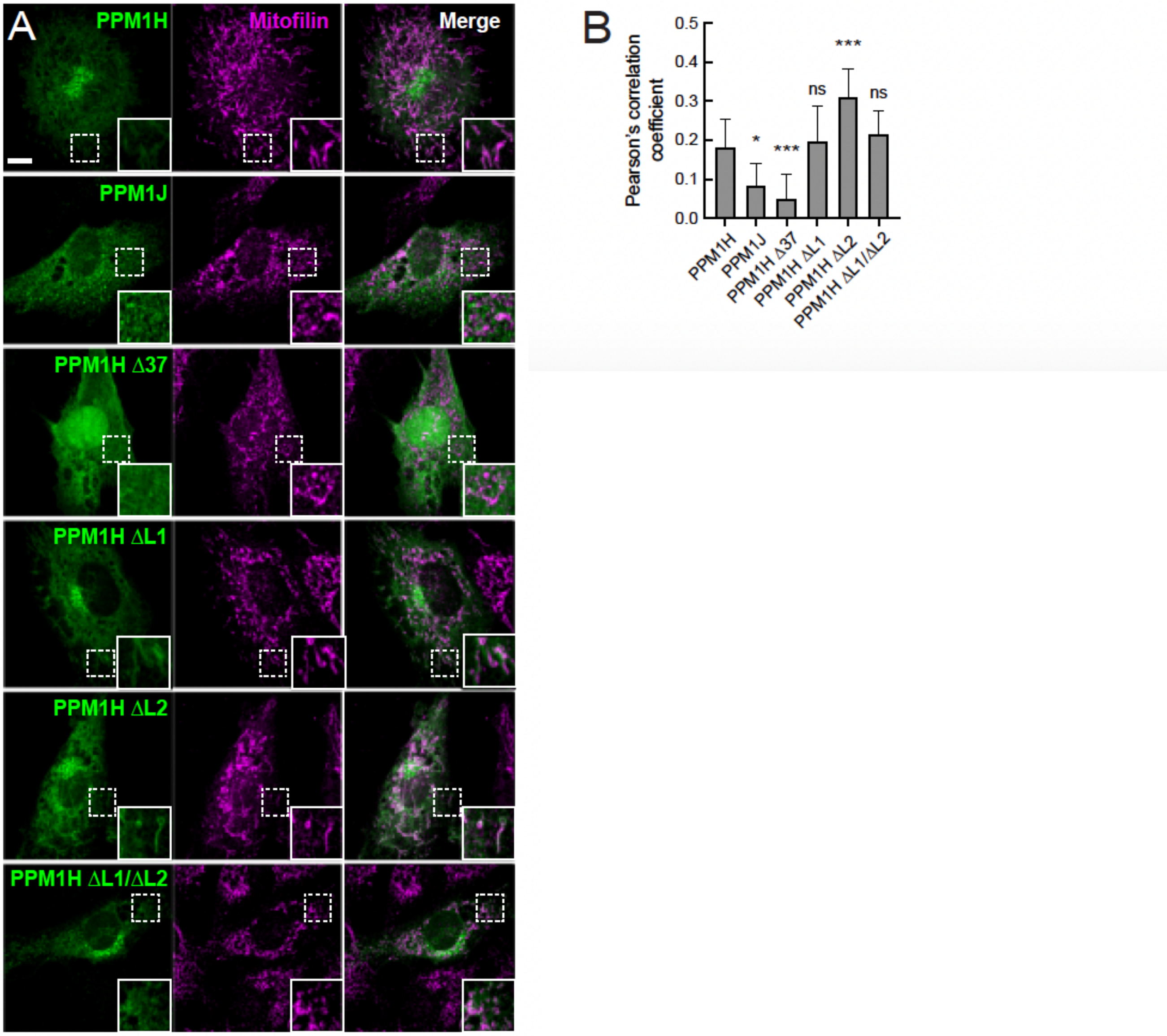

Fig. S2. PPM1H constructs are mostly non-mitochondrial in RPE cells.
RPE cells were transiently transfected with plasmids encoding either HA-PPM1H, HA-PPM1J, HA-Δ37-PPM1H, HA-ΔLoop1-PPM1H (missing 115-133), HA-ΔLoop2-PPM1H (missing 204-217) or HA-ΔLoop1/2-PPM1H (missing both loops). After 24 hours cells were subsequently fixed and stained with mouse anti-HA antibody (green) and rabbit anti-mitofilin (red). Scale bar, 10 μm. Shown are maximum intensity projections. Areas boxed with dashed lines are enlarged at lower right. (B) Co-localization of PPM1H or PPM1J with mitofilin was determined by Pearson's coefficient. Error bars represent SEM from two independent experiments in which more than 20 cells per condition were analyzed. Significance was determined relative to HA-wtPPM1H by one way ANOVA. *p=0.010169 for HA-wtPPM1H and HA-wtPPM1J, ***p=0.000339 for HA-wtPPM1H and HA-Δ37PPM1H, p=0.986811 for HA-ΔLoop1PPM1H, ***p=0.000202 for HA-wtPPM1H and HA-ΔLoop2PPM1H, p=0.628636 for HA-wtPPM1H and HA-ΔLoop1/2PPM1H.

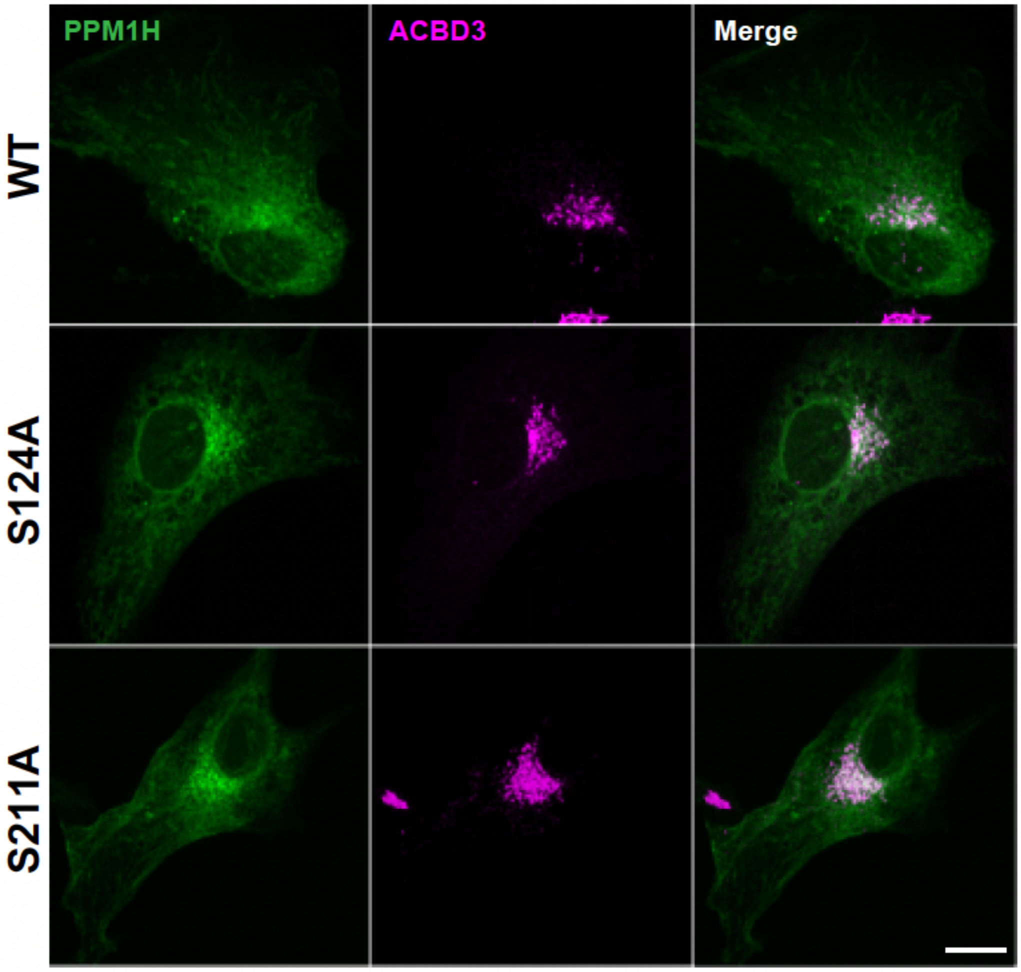

Fig. S3. PPM1H phosphorylation mutants localize to the Golgi complex. The indicated PPM1H constructs were transfected and evaluated as in Figure 1. Scale bar, 10μm.

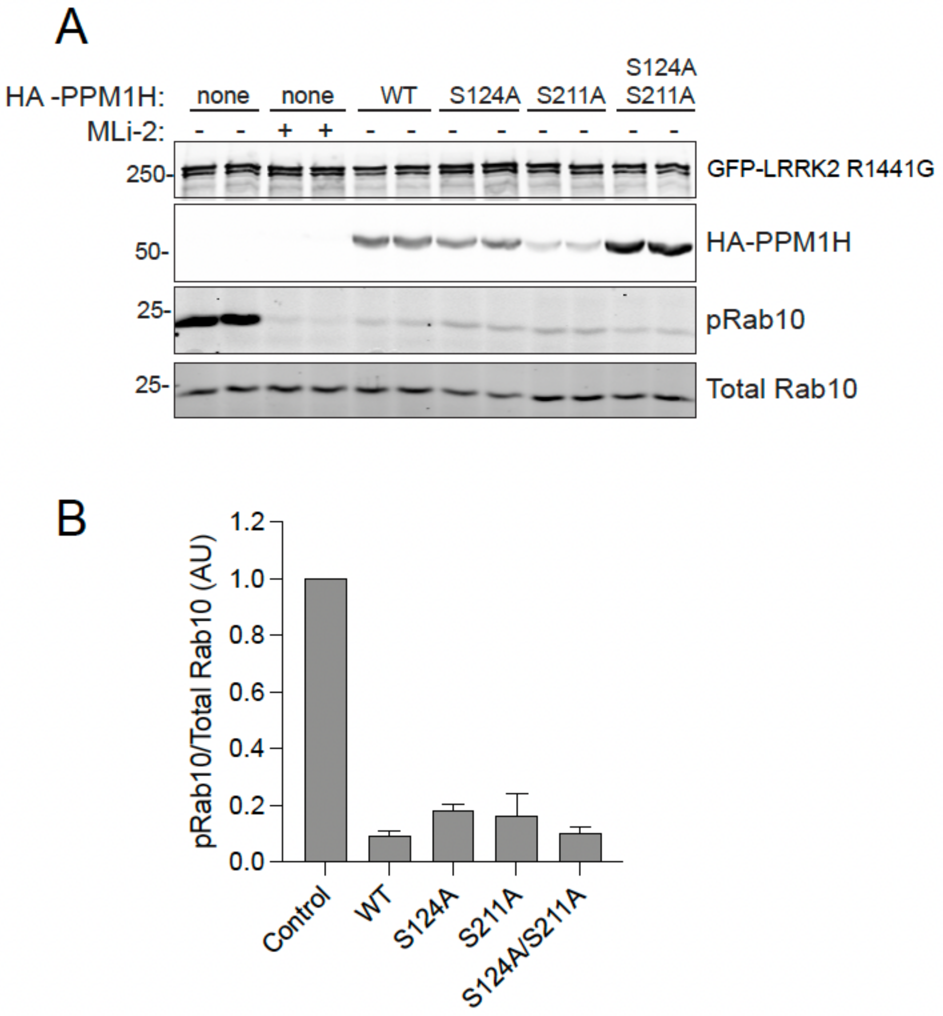

Fig. S4. PPM1H phosphorylation site mutants are fully active. (A) HEK293T cells were transiently transfected for 24h with GFP-LRRK2 (R1441G) together with either HA-empty, HA-PPM1H, or HA-PPM1H S124A, S211A, or S124A/S211A. After 24 h cells were lysed and 30 µg protein analyzed by immunoblotting. Proteins were detected using mouse anti-LRRK2, rabbit anti-HA, rabbit anti-pRab10, and mouse anti-total Rab10 antibodies. (B) Activity of PPM1H was quantified as the ratio of pRab10 to total Rab10. Error bars represent SD from two independent experiments.

Key Resources Table				
**Reagent type (species) or resource**	**Designation**	**Source or reference**	**Identifiers**	**Additional information**
Antibody	anti-LRRK2 (mouse monoclonal)	Neuromab	N241A/34 (RRID:AB_10675136)	(1:1000)
Antibody	anti-Rab10 (mouse monoclonal)	Nanotools	0680–100/Rab10-605B11 (RRID:AB_2921226)	(1:1000)
Antibody	anti-Rab10 (phospho T73) (rabbit monoclonal)	Abcam	Ab230261 (RRID:AB_2811274)	(1:1000)
Antibody	anti-PPM1H (Sheep polyclonal)	MRC-PPU	F-1804 (RRID:AB_2923281)	(1:1000)
Antibody	anti-PPM1H (rabbit monoclonal)	Abcam	ab303536 (RRID:AB_2941812)	(1:1000)
Antibody	anti-Rab12 (phospho S106) (Rabbit monoclonal)	Abcam	Ab256487 (RRID:AB_2884880)	(1:1000)
Antibody	anti-Rab12 (mouse monoclonal)	Santa Cruz Bio	sc-515613 (No RRID available)	(1:500)
Antibody	chicken anti-GFP (Chicken polyclonal)	Aves	AB_2307313 (RRID:AB_10000240)	(1:2000)
Antibody	anti-α-Tubulin (mouse monoclonal)	Santa Cruz Bio	sc-5286 (RRID:AB_628411)	(1:1000)
Antibody	Anti-mitofilin (Rabbit Polyclonal)	Novus	NB100-1919 (RRID:AB_2127207)	(1:250)
Antibody	anti-HA (mouse monoclonal)	Sigma	H9658 (RRID:AB_260092)	(1:500)
Antibody	Anti-ACBD3 (Rabbit Polyclonal)	Sigma	HPA015594 (RRID:AB_1844491)	(1:1000)
Antibody	anti-HA (Rabbit monoclonal)	CST	3724 (RRID:AB_1549585)	(1:1000)
Antibody	anti-LAMP2 (mouse monoclonal)	DSHB	H4B4 (RRID:AB_2134755)	(1:1000)
Antibody	anti-myc (mouse monoclonal)	BioLegend	626802 (RRID:AB_2148451)	(1:1000)
Antibody	H+L Donkey anti-mouse Alexa 488	Life Technologies	A32766 (RRID:AB_2762823)	
Antibody	H+L Donkey anti-mouse Alexa 568	Life Technologies	A10037 (RRID:AB_2534013)	
Antibody	H+L Donkey anti-Rabbit Alexa 488	Life Technologies	SA5-10038 (RRID:AB_2556618)	
Antibody	H+L Donkey anti-Rabbit Alexa 568	Life Technologies	A10042 (RRID:AB_2534017)	
Antibody	680 nm donkey anti rabbit secondary antibodies	Li-COR	926-68073 (RRID:AB_10954442)	
Antibody	680 nm donkey anti mouse secondary antibodies	Li-COR	926-68072 (RRID:AB_10953628)	
Antibody	800 nm donkey anti rabbit secondary antibodies	Li-COR	926-32213 (RRID:AB_621848)	

Antibody	800 nm donkey anti mouse secondary antibodies	Li-COR	926-32212 (RRID:AB_621847)	
Antibody	680 nm donkey anti Chicken secondary antibodies	Li-COR	926-68075 (RRID:AB_10974977)	
bacterial strain	E. coli DH5α	Thermo Fisher	18258012	
bacterial strain	E. coli STBL3	Thermo Fisher	C737303	
bacterial strain	E. coli Rosetta DE3 pLys	Millipore	70956	
cell line	RPE	ATCC	CRL-2302 (RRID:CVCL_0145)	
cell line	HEK293T	ATCC	CRL-3216 (RRID:CVCL_0063)	
Cell line	A549	ATCC	ATCC-CCL-185 (RRID:CVCL_0023)	
Cell line	A549 PPM1H KO	MRC PPU	PMIID:31663853	
Chemical compound, drug	MLi-2	MRC PPU	CAS no.: 1627091-47-7	
Filter	Drain disc 10mm	Sigma Aldrich	WHA230300	
Filter	Polycarbonate membranes 0.05mm	Avanti Polar Lipids Inc.	610003	
Filter	Polycarbonate membranes 0.1mm	Avanti Polar Lipids Inc.	610005	
Filter	Polycarbonate membranes 0.4mm	Avanti Polar Lipids Inc.	610007	
Lipid	18:0-20:4 PC	Avanti Polar Lipids	#850469	
Lipid	18:0-20:4 PI	Avanti Polar Lipids	#850144	
Lipid	18:1 PI(4)P	Avanti Polar Lipids	#850151	
Lipid	18:0-18:2 PS	Avanti Polar Lipids	#840063	
Lipid	Cholesterol	Avanti Polar Lipids	#700016	
plasmid	pcDNA 3.1 myc-Rab10	Addgene	RRID: Addgene_208366	human
plasmid	pcDNA 3.1 myc-Rab10-mito	Addgene	RRID: Addgene_208367	human
plasmid	pCMV5D HA-PPM1H	MRC PPU	DU62789	human
plasmid	pCMV5D HA-PPM1H S124A	Addgene	RRID: Addgene_208368	human
plasmid	pCMV5D HA-PPM1H S211A	Addgene	RRID: Addgene_208369	human
plasmid	pCMV5D HA-PPM1H S124A/S211A	Addgene	RRID: Addgene_208370	human
plasmid	pCMV5D HA-Δ10 PPM1H	Addgene	RRID: Addgene_207335	human
plasmid	pCMV5D HA-Δ20 PPM1H	Addgene	RRID: Addgene_207336	human
plasmid	pCMV5D HA-Δ34 PPM1H	Addgene	RRID: Addgene_207337	human

plasmid	pCMV5D HA-Δ37 PPM1H	Addgene	RRID: Addgene_207338	human
plasmid	pCMV5D HA-Δ44 PPM1H	Addgene	RRID: Addgene_207339	human
plasmid	pCMV5D HA-Δ71 PPM1H	Addgene	RRID: Addgene_207340	human
plasmid	pCMV5D HA-L2D/V9D/I19D PPM1H	Addgene	RRID: Addgene_207341	human
plasmid	pCMV5D HA-Δ115-133, 204-217 PPM1H	Addgene	RRID: Addgene_207343	human
plasmid	pCMV5D HA-Δ37, 115-133, 204-217 PPM1H	Addgene	RRID: Addgene_207344	human
plasmid	pCMV5D HA-PPM1J	MRC PPU	DU68077	human
plasmid	pET15b+His-SUMO PPM1H	Addgene	RRID: Addgene_207333	human
plasmid	pET15b+His-SUMO Δ37 PPM1H	Addgene	RRID: Addgene_207334	human
plasmid	pET15b+His SUMO Rab10 (1-181) Q68L	Addgene	RRID: Addgene_207342	human
Plasmid	CSII-PPM1H-mApple	Addgene	RRID: Addgene_198473	human
plasmid	pCMV5 FLAG-LRRK2	MRC PPU	DU6841	human
plasmid	pCMV5 Flag-LRRK2 R1441C	MRC PPU	DU13078	human
Plasmid	pMCB306-GFP-Rab12	Addgene	RRID: Addgene_198471	human
Plasmid	pMCB306-GFP-Rab10	Addgene	RRID: Addgene_130883	human
Plasmid	pMCB306-GFPRab8	Addgene	RRID: Addgene_198470	human
Plasmid	pMCB306-GFP-Rab29	Addgene	RRID: Addgene_198472	human
Plasmid	pET 15b+His SUMO Rab12 (Q101L)	Addgene	RRID: Addgene_208371	human
Plasmid	Lenti-TO-Δ37-HA PPM1H	Addgene	RRID: Addgene_208372	human
Plasmid	Lenti-TO-Δ37 PPM1H -PACT-HA	Addgene	RRID: Addgene_208373	human
Plasmid	Lenti-TO-Δ37 PPM1H-TMEM115-HA	Addgene	RRID: Addgene_208374	human
Plasmid	Lenti-TO-HA-Δ37 PPM1H-mito	Addgene	RRID: Addgene_208375	human
Plasmid	pmcb306-GFP-Rab12-mito	Addgene	RRID: Addgene_207349	human
Plasmid	CSII-mApple	Addgene	RRID: Addgene_207350	human
Plasmid	CSII-PPM1H-mApple-mito	Addgene	RRID: Addgene_207351	human

Software, Algorithm	FIJI/ImageJ	PMID: 29187165	RRID:SCR_002285	
Software, Algorithm	ImageJ	https://imagej.net/	ImageJ RRID:SCR_003070	
Software, Algorithm	CellProfiler	PMID: 29969450	RRID:SCR_007358	
Software, Algorithm	Chimera X	PMID:32881101	RRID:SCR_015872	
Software, Algorithm	Prism	Prism 9 version 9.3.1 (350)	RRID:SCR_002798	
Software, Algorithm	Heliquest	PMID: 18662927	https://heliquest.ipmc.cnrs.fr/	
